# List of Cases Admitted under the Care of the Physicians of the Universal Dispensary for Children, St. Andrew's Hill, Doctors' Commons, London; from the 31st of October, 1817, to the 31st of December Following

**Published:** 1818-04

**Authors:** J. B. Davis, Wm. Shearman

**Affiliations:** Physicians; Physicians


*List of Cases admitted under the Care of the Physicians of the Universal Dispensary for Children, St. Andrew's Hill, Doctors' Commons, London; from the* 31*st of October,* 1817, *to the* 31*st of December following.*

| Diseases. | Cases. | Diseases. | Cases. |
|---|---|---|---|
| Febris | 19 | Cynanche Parotidœa | 1 |
| —— Remittens | 10 | —— Tonsillaris | 2 |
| —— Tertiana | 3 | —— Trachealis | 9 |
| Febricula | 12 | Epilepsia | 2 |
| Pneumonia | 11 | Scarlatina | 5 |
| Dysenteria | 10 | Varicella | 2 |
| Diarrhœa Crapulosa | 12 | Rachitis | 9 |
| —— Mucosa | 14 | Hydrocephalus Acutus | 7 |
| —— Sanguinea | 11 | —— Chron. | 8 |
| Cholera Morbus | 3 | Paralysis | 2 |
| Morbilli | 24 | Epistaxis | 1 |
| Pertussis | 42 | Chorea Sancti Viti | 2 |
| Erysipelas | 5 | Vermes Ascarides | 9 |
| Tormina | 8 | —— Lumbrici | 3 |
| Phrenitis | 12 | —— Tænia | 2 |
| Convulsiones | 10 | Crusta Lactea | 2 |
| Tussis | 18 | Dyspepsia | 14 |
| Icterus | 12 | Urticaria | 4 |
| Tabes Mesenterica | 8 | Psoriasis | 2 |
| Roseola | 2 | Enteritis | 8 |
| Dyspnœa | 4 | Aphthæ | 5 |
| Febris Catarrhalis | 12 | | |
| Erythema | 12 | | 383 |
| Eruptiones Variæ | 10 | | |

---

The Records of the prevailing Diseases among the Children admitted into the Universal Dispensary for Children, during the last year, contained in the Medico-Chirurgical Journal, show that Influenza, in 1817, was a very predominant, and likewise a very fatal malady. In the months of October, November, and December, of 1817, this disease, on the contrary, has scarcely been seen. Catarrhal affections have appeared in their ordinary form, at this season; but they seldom were severe, and still less frequently did they end in Pneumonia.

Synocha has been very prevalent among children of three, four, and five years of age, and upwards. The head has invariably been affected with considerable pain; the eye heavy, the pupil dilated. In some of the cases, the eye was extremely bright and the conjunctiva red: in the latter, the pyrexia was more intense. During the months of October, November, and December, Synocha has been the reigning fever; Typhus, and the bilious remitting fever of the autumn having nearly disappeared. Genuine Pneumonia was of frequent occurrence. The children who died of it had extensive adhesions of the lungs to the pleura; and in some of the dissections, pus was found between the pleura and the lungs. Measles have, in general, been mild; but hooping-cough severe. The latter was, in many instances, conjoined with Pneumonia; and effusion into the bronchiæ by no means rare.

Eruptive fevers have been few and mild; the Croup has occurred with severity in three or four children. Hydrocephalus Acutus and Chronicus as usual, in their several stages, made a prominent feature in the list of the diseases which were admitted within the period of the present Report.*

J. B. DAVIS,<br>WM. SHEARMAN, } Physicians.

---

* It is intended to give these Reports quarterly in future, and to publish them regularly, at an earlier period after the books are made up.

PART

